# Healer-led vs. clinician-led training to improve personal protective equipment use among traditional healers in South Africa: a randomized controlled trial protocol

**DOI:** 10.1080/16549716.2021.1898131

**Published:** 2021-04-02

**Authors:** Carolyn M. Audet, Bryan E. Shepherd, Muktar H. Aliyu, Mosa Moshabela, Mariah J. Pettapiece-Phillips, Ryan G. Wagner

**Affiliations:** a Vanderbilt Institute for Global Health, Vanderbilt University Medical Center, Nashville, TN, USA; b Department of Health Policy, Vanderbilt University Medical Center, Nashville, TN, USA; c MRC/Wits Rural Public Health and Health Transitions Research Unit (Agincourt) School of Public Health, Faculty of Health Sciences, Johannesburg, South Africa; d Department of Biostatistics, Vanderbilt University Medical Center, Nashville, TN, USA; e School of Nursing and Public Health, College of Health Sciences, University of KwaZulu Natal, Durban, South Africa

**Keywords:** Personal protective equipment, traditional healers, HIV prevention, randomized controlled trial, blood exposure

## Abstract

There are estimated two million traditional healers in sub-Saharan Africa (SSA), with more than 10% (200,000) working in South Africa. Traditional healers in SSA are frequently exposed to bloodborne pathogens through the widespread practice of traditional ‘injections’, in which the healers perform dozens of subcutaneous incisions to rub herbs directly into the bloodied tissue with their hands. Healers who report exposure to patient blood have a 2.2-fold higher risk of being HIV-positive than those who do not report exposure. We propose a randomized controlled trial (61 healers in the intervention group and 61 healers in the control group) in Mpumalanga Province. Healers will receive personal protective equipment (PPE) education and training, general HIV prevention education, and three educational outreach visits at the healer’s place of practice to provide advice and support for PPE use and disposal. Healers in the control arm will be trained by health care providers, while participants in the intervention arm will receive training and outreach from a team of healers who were early adopters of PPE. We will evaluate intervention implementation using data from surveys, observation, and educational assessments. Implementation outcomes of interest include acceptability and feasibility of PPE use during clinical encounters and fidelity of PPE use during treatments that involve blood exposure. We will test our two intervention strategies to identify an optimal strategy for PPE education in a region with high HIV prevalence.

## Background

In rural South Africa, traditional healers provide physical and psychological services to >80% of the population [1]. There are more than 2 million traditional healers in sub-Saharan Africa (SSA), 200,000 of whom work in South Africa [2–6]. A patient often first seeks a healer for their health needs, receiving herbal remedies (‘injected’/incised into the skin and ingested) to prevent and cure ailments [7]. While substantial research efforts have been expended on understanding the positive [8–17] and negative [4,18,19] impact that healers can have on the health of their patients, occupational hazards associated with traditional healer practices in SSA have attracted scant attention to date [19]. Allopathic health care workers are recognized to be at risk for HIV, Hepatitis B virus (HBV), Hepatitis C virus (HCV), and other bloodborne infections through occupational exposure to blood and body fluids [20–24].

Similar to allopathic health care workers, healers are also exposed to patients’ blood and body fluids. A widespread practice is the traditional ‘injection,’ (described as such by traditional healers but could more accurately be called incisions) in which the healer performs dozens of subcutaneous incisions in order to rub herbs directly into the bloodied skin [25–27]. In South Africa, 98% of healers report conducting ‘injections’, resulting in estimated 1,500 blood exposures over the course of their lifetime [7]. Despite pressure to eliminate the practice of ‘injections,’ these treatments have been used for centuries and are often the preferred and expected treatment delivery method among patients [28,29].

Training in the donning, doffing, and disposal of personal protective equipment is known to have a positive impact on correct PPE use among health care providers [30–36] although training has primarily been led by expert clinicians. These in-person training programs are essential to reducing the risk of infection among providers. Recently, peer-led training to improve clinical skills or service delivery has shown equal or greater impact than traditional ‘expert-led’ training programs [37,38]. Peer-led training can increase intrinsic motivation, alleviate health care provider teaching burden, and provide accessible role models for learners [39]. We propose to test the impact of a peer-led PPE training model (vs. training led by expert health care providers) to increase correct use (donning, doffing, and disposal) by traditional healers living in Bushbuckridge, South Africa.

## Methods

### Study rationale

#### The use of PPE among traditional healers

Traditional healers risk acquiring bloodborne pathogens from contaminated blood and body fluids. The risk of infection varies by pathogen, type of exposure, amount of blood involved, and amount of virus in the patient’s blood at the time of exposure [40]. The average risk of HIV infection after a needlestick or cut exposure to HIV-infected blood is 0.3%, and after exposure to eye, nose, or mouth is estimated to be 0.1% [40]. ‘Injections’ result in substantial blood exposure: approximately 3 mL of blood per injection, directly in contact with a healer’s bare skin (primarily the hands and arms, though the average person touches their face 23 times per hour, thus exposure to the mucous membranes is possible) [41]. Many healers are subsistence farmers; planting and harvesting often cause small cuts and abrasions on their hands, worsened by regular household activities, including food preparation and laundry. Limited access to potable water (thus, limited handwashing opportunities post-‘injection’) exacerbates the risk of acquisition. In Bushbuckridge, Mpumalanga, healers have an overall HIV prevalence of 30% (CI: 23%-37%) compared to 19% in the general population [42]. Healers in this region who reported exposure to patient blood had a 2.4-fold increased risk of being HIV-positive compared to those with no reported blood exposure (59% vs. 25%, respectively) [7].

Low rates of personal protective equipment (PPE) training and use place healers at risk. PPE is defined as equipment or instruments (gloves, safety glasses, facemasks, respirators, etc. [43]) worn to avoid and/or minimize exposure to hazards that could cause injuries or the spread of infection and illness. The use of PPE has been shown to be effective in reducing occupational exposures [43–45]. Similar to clean needle exchange programs, the provision of PPE should not enable traditional ‘injection’ behavior. Instead, it bridges the divide between these two systems and reduces healers’ exposure to occupational hazards. Strategies implemented in healthcare settings to increase PPE use include educating healthcare workers (HCWs) about infection risk, as well as the provision, training, and use of PPE. However, strategies specifically aimed at increasing uptake of PPE among traditional healers and other community health workers are underexplored. Traditional healers are aware that there are risks associated with exposure during invasive procedures, but few purchase PPE or use it correctly [46].

### Design

**Figure 1.**
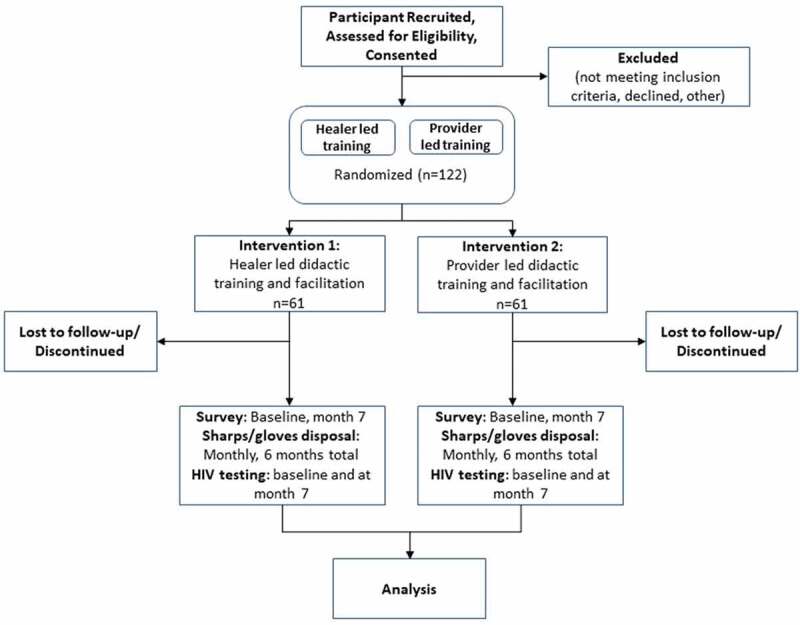
Trial Profile

We propose to test the impact of two implementation strategies on healer blood exposure via a type II hybrid effectiveness-implementation individually randomized controlled trial [47]. A type II trial is one that focuses equally on implementation outcomes (fidelity to training) and effectiveness (use of PPE during every blood exposure). We will assess the impact of two implementation strategies on healer blood exposure via an individually randomized controlled trial of 122 healers (61 in each arm) from Bushbuckridge, Mpumalanga, South Africa. We have selected a comparison of two implementation strategies to deliver an evidence-based intervention to traditional healers for ethical and practical reasons. We believe all healers should have access to quality training; if healers can provide services with equal fidelity it makes scaling up training nationwide more feasible. We will randomize traditional healers into: (1) a control arm, which will receive didactic training led by clinicians on PPE use, general HIV prevention education and skill-building, as well as implementation facilitation and (2) the intervention arm, which will receive the same didactic training and implementation facilitation led by traditional healers who were early adopters of PPE (Figure 1). To avoid concerns that, despite randomization, one intervention arm of healers will substantially differ from the other, we will conduct stratified randomization. Specifically, we will stratify based on healer sex and the number of patients seen in a given month (dichotomized into high/low based on the estimated median number of patients seen in the past month). This will result in 4 strata. At the time of randomization, each healer will be put in a strata and equal numbers of healers in each strata will be randomized to the two intervention arms.

### Aims

1. Evaluate the fidelity with which early adopter healers and clinicians provide didactic PPE training and implement facilitation and mentorship to healers.
2. Compare the clinical effectiveness of the two intervention strategies. Are traditional healers in the intervention arm more likely to use PPE regularly and correctly during treatments that could result in blood exposure than those in the control arm?

### Study setting

Vanderbilt University Medical Center and the University of the Witwatersrand have partnered to implement this research. The study will be conducted in the rural Bushbuckridge sub-district of Mpumalanga province, South Africa. The Medical Research Council/Wits Agincourt Research Unit oversees the maintenance and operation of the Agincourt Health and Demographic Surveillance Site (HDSS). Roughly 500 km northeast of Johannesburg, the unit has been engaged in population-based health- and socio-demographic research since 1992. Strong ties with the local community ensure the continual functioning and sustainability of the research. The Agincourt HDSS population is comprised of roughly 120,000 mainly xiTsonga-speaking individuals spread throughout 20,000 households in 31 research villages that are surveyed annually. The HDSS includes all individuals living in the region, regardless of their immigration or economic status, tracks all in- and out-migration, and includes a verbal autopsy component to assess any deaths. Being a former homeland during Apartheid the Agincourt area is generalizable to much of South Africa in terms of its infrastructure (or lack thereof), its population’s socio-demographic characteristics (including levels of formal education, migration and socio-economic status) as well as disease burden and health care system. Our community links and continuing presence make Agincourt a uniquely well-suited site (due to existing infrastructure, research support, and proven track record) for testing this intervention

### Ethics

The study has been approved by the Vanderbilt University Institutional Review Board and the University of Witwatersrand Human Research Ethics Committee (Medical). Before enrolling any participants, a detailed consent explaining the purpose of the study including its goals, the procedures associated with enrollment into the study, the risks and benefits of participation, the voluntary nature of this study, how the findings from this study will be disseminated/published including details on participant confidentiality, as well as information for study participants to use if they need to contact anyone about their rights as a research participant and/or they have questions for the study coordinators, and/or the study Principal Investigator will be provided. Each potential participant will be informed that participation in the study is completely voluntary and that he or she may withdraw from the study at any time. Included in this informed written consent will be detailed pertaining to the existing standard of care for delivering information about blood exposures/PPE to traditional healers. Also included in this consent will be language explaining that the results from this study will be made available to the public in manuscript format (presenting only completely de-identified data/results).

### Eligibility criteria

#### Inclusion criteria

1. Traditional healers ≥18 years of age, who are registered as traditional healers with the government of South Africa, are currently practicing in the Bushbuckridge area, and conduct traditional injections.
2. Biomedical practitioners ≥18 years of age, who are currently providing health care services to patients at government or private health facilities in Bushbuckridge.

#### Exclusion criteria

1. Traditional healers <18 years of age, who are not registered as traditional healers with the government of South Africa, are not currently practicing, or do not conduct traditional injections on their patients.
2. Biomedical practitioners <18 years of age or who are not currently providing health services in the Bushbuckridge area.

### Subject recruitment

#### Selection of six early adopter traditional healers

Traditional healers are considered early adopters if they report using PPE at every patient encounter that may involve exposure to patient blood (e.g. during ‘injections’). A cohort of individuals who fit these criteria has been established but recruitment of potential trainers has not yet begun. We will provide interested healers the adapted PPE training using a train-the-trainer model [48] to ensure that our early adoptor healers are both equipped and comfortable providing PPE training to other healers.

#### Selection of health care worker trainers

Health care workers will be recruited if they provide primary care or HIV/TB services in the primary health care facility. A list of eligible providers will be elicited from the facility chief; one meeting to discuss the study will be held in the clinic. If the health care worker is interested in participating, they will be consented by a study assistant at the health facility or in a location of their preference.

#### Selection of participant traditional healers

Healers will be recruited through two means: (1) Healers in target catchment area are currently being mapped by two medical students (one from Vanderbilt and one from the University of Witwatersrand). The healers are providing their address and contact information (phone numbers, contact for other family members) for use in future activities (including research endeavors). The healers will be contacted and given general information about the proposed study. All healers will be invited to community meetings where more information about the study procedures and aims will be provided. (2) The Kukula Traditional Healers association (see letter of support)- the primary healer organization in the region- will hold 3 meetings to recruit healers interested in participating. Healers who express interest will be given the option for dates/locations where we will conduct recruitment fairs.

### Intervention

Didactic training: In 2010, the CDC developed guidelines and a short course to train healthcare personnel on selecting and using PPE correctly [49]. Additional PPE training programs from the WHO and the Occupational Safety and Health Administration (OSHA) include courses on: (1) bloodborne pathogens, (2) hazard communication, and (3) PPE [50]. The programs include interactive presentations aimed at nurses, physicians, technicians, and ancillary personnel in healthcare (e.g. housekeepers, maintenance workers) in the US. The training is divided into didactic presentations, question and answer sessions, PPE demonstrations, and simulated patient treatment. We will adapt, in collaboration with PPE trainers at the University of the Witwatersrand, Charlotte Maxeke Johannesburg Academic hospital, Tintswalo Hospital, and PPE trainers from several smaller clinics in Agincourt, the PPE training course to ensure it is localized, feasible, and appropriate for traditional healers. The course material will cover, at a minimum, the following elements: epidemiology, symptoms, and transmission of bloodborne pathogens; what constitutes a BBF exposure; universal precautions for preventing transmission of bloodborne pathogens; work practice controls; hepatitis B vaccine, including information on its efficacy, safety, method of administration, and the benefits of being vaccinated; types, uses, removal, handling, decontamination, and disposal of PPE; management of regulated waste; sharps safety; and post-exposure evaluation, including linkage to care, etc. Simulated patient care has been linked to increased PPE compliance [51]. At the end of the training, a traditional healer will be able to: (1) identify appropriate circumstances in which PPE is indicated and the risk posed to a healer’s health; (2) list at least three safe practices to follow when using PPE; (3) correctly demonstrate how to don and doff PPE.

Facilitation: In addition to the short course training, each participant will receive three facilitation visits at the healer’s place of practice to provide on-the-job advice and practical support for PPE use and disposal. All participants will be provided PPE (gloves and masks), as well as medical sharps and medical waste containers for used razor blades and gloves.

### Outcomes and primary comparisons

2.9.1 Aim 1: We will rate training fidelity via two constructs: (1) content and (2) coverage [52,53] during the week-long training session and community-based outreach sessions (see Appendix materials). Understanding the fidelity of training delivery is essential to interpreting the effect of the intervention on PPE behavior change. If HCW + healer teams can deliver training with greater or equal fidelity than HCWs alone, PPE training can be more quickly scaled up nationwide (given the large number of healers) and conducted at lower cost (healers charge considerably less per hour than HCWs) [54]. We believe that the healer + HCW teams may have more motivation to deliver the program with fidelity during community-based sessions than the team of HCWs alone because of healers’ desire to protect their peers living in their own community. Three HCWs who volunteer to work on this project will be randomly assigned to the intervention team (along with the healers), with six volunteer HCWs assigned to the control arm.

2.9.2 Aim 2: During the 6 months of the trial, healers in each arm will record data on our primary outcome: the proportion of injections conducted using PPE in two ways. (1) Collect used sharps and PPE. Healers will be given containers to place all used gloves and razors/sharps to both ensure healer safety and allow us to count materials used (healers use gloves and a single razor once per patient and subsequently throw them into latrines or trash piles). We will use the number of glove pairs as the numerator (number of times they used gloves during procedures) and the number of razor blades as the denominator (number of injections given). While some may be concerned with social desirability bias, healers have been told for years to use gloves and were very open with us about their non-use. Healers have no reason to under- or over-inflate their PPE use as it will not benefit them; in fact, they would have to buy the razor blades or travel to a health facility to obtain additional latex gloves, something that seems unlikely in this population. (2) Self-report. Healers will record behavior (injection and glove use) every time they conduct an injection. In addition to collecting used PPE and sharps, we will also conduct brief interviews with traditional healers about their use of PPE multiple times or the use of only a single glove during a visit to stretch their PPE resources and administer a brief social desirability measure to determine if they feel compelled to inflate their PPE use [57].

### Analysis plan

#### Evaluation of implementation fidelity

Analysis plan for content: A numeric scale will be used to assess the fidelity of the delivery of *each* topic in the training session. Topics include the delivery of: (1) Information about the definition of PPE, (2) Information about when to use PPE, (3) Information about what types of PPE to use in different circumstances, (4) Information about the appropriate fit of PPE, (5) Information about the do’s and don’ts of glove use, (6) Information about the donning of PPE, (7) Information about how and where to remove PPE, (8) Information about how to safely store used PPE, (9) Information about hand hygiene, and (10) Instruction and feedback during the PPE practice session. Each of the 10 training items will be scored (each with a score of 0–10, where 10 represents the greatest fidelity to the training guide, and 0 denotes not covering the material at all), for a total of 100 points [56]. The summation of all scores across topics will be used to measure fidelity to content for each training session. Each participant will participate in three training sessions.

The mean difference between the two interventions in the total fidelity scores will be estimated using mixed effects linear regression models with 95% confidence intervals (CIs). These models will include random effects for the participant (to account for correlation between scores from the same participant across multiple sessions) and the trainer (to account for correlation between scores from different participants who are instructed by the same trainer) and a 95% CI will be constructed. The outcome will be transformed as necessary to meet modeling assumptions. More specifically, QQ-plots of model residuals will be examined to assess whether normality assumptions are reasonable; if not, we will consider log10 or Box-Cox transformations, and compare the sensitivity of results to no transformation. Covariates will include patient volume, sex, and baseline age.

Data analysis will be conducted using R. Any (>0%) missing data will be multiply imputed using chained equations with 20 imputation replications; complete-case analyses (i.e. excluding records with missing data) and best-worst case analyses (i.e. imputing missing data with extremely low and extremely high values) will be performed as sensitivity analyses [58]. Based on prior experience working with healers, we anticipate very low rates of loss to follow-up. Healers who are lost to follow-up will be censored at the time of their last recorded data collection, and inverse probability of censoring weights based on healer characteristics at randomization will be used, if necessary, to account for differences between healers who are lost to follow up and those who remain in the study.

Analysis plan for coverage: To calculate the percentage of sessions completed, we will divide each participant’s actual number of sessions completed by the total number of planned sessions. We will compare the percentage of sessions completed between the two intervention arms using mixed effects Poisson regression with the number of planned sessions as an offset. 95% confidence intervals will be constructed. The models will include random effects for the trainers. Covariates will include patient volume, sex, and baseline age. Missing data and loss to follow-up will be handled as described earlier.

#### Analysis plan for effectiveness

The analysis will follow intent-to-treat protocol. We will compare the blood exposure proportions between the two study arms using a negative binomial regression model with the outcome being the number of times gloves were used. The number of procedures performed by the healer (number of razor blades) will be included as an offset. This analysis is appropriate as it properly accounts for heterogeneity between healers in the number of procedures performed, and the resulting estimates from the model can be interpreted as relative proportions (proportion of procedures with latex gloves in Arm 1 vs. proportion of procedures with latex gloves in Arm 2). In addition to our primary intervention exposure variable, we will include healer sex and baseline age as covariates in our model. Sensitivity analyses will compare the distribution of latex glove proportions between the two intervention arms using a Wilcoxon rank sum test. We will compare the results of our blood exposure methods (materials vs. self-report). If there are significant differences, we will conduct qualitative interviews to determine which one is more accurately estimating risk behavior.

A separate analysis will assess each strategy’s effect on PPE knowledge, motivation, skills, and self-efficacy post-training [55] (see Appendix for details). We will explore whether the intervention’s effect on these measures differ by trainer fidelity to the intervention and/or trainer identity (healer vs. clinician). These analyses will provide quantitative data to revise and refine, as needed, the intervention for evaluation in a large RCT effectiveness study as a means of improving prevention engagement while reducing stigma. Lastly, we will perform mediation analyses to assess whether these secondary endpoints are mediated by implementation factors, including acceptability of PPE, pressure from other healers to use PPE, and the success of the implementation process. Mediation analyses will follow accepted analytical approaches. The variable transformation will be considered to meet modeling assumptions.

#### Power and sample size

From preliminary data collected from healers in 2019, the median proportion of glove use during injections was 0.44, mean 0.39, and standard deviation (SD) 0.39. Our sample size is calculated based on a two-sample t-test and incorporates these preliminary data. Assuming a standard deviation of 0.39 in both arms, we anticipate needing 61 healers per arm (total of 122) to have 80% power to detect (at the alpha = 0.05-level) a difference of 0.2 in the proportion of glove use between HCWs and healer teams (e.g. 0.4 vs. 0.2) (or equivalently a standardized mean difference of 0.51).

## Discussion

Traditional healers are at high risk of contracting HIV and other blood-borne pathogens during patient encounters that often involve contact with patient’s blood. Effective strategies to implement evidence-based PPE training programs among non-clinical workers are yet to be established. Testing these strategies with traditional healers in rural South Africa is the first step to a large-scale trial in sub-Saharan Africa, where more than 2 million healers' practice. The protocol described was developed in partnership with traditional healers in Agincourt, South Africa, and will employ rigorous methods to identify and address multilevel barriers to sustainability of PPE training and use. We are unable to implement a fully powered trial to assess healer seroconversion, given the financial and temporal limitations of an R21 award. However, there is substantial evidence linking blood exposure to HIV infection; thus, we believe our proxy measure is appropriate. If we detect a significant decrease in blood exposure, we will propose a definitive RCT (R01) to assess the impact of PPE interventions on HIV seroconversion among a large representative group of healers living across South Africa to determine whether PPE training can reduce HIV incidence among healers.

## Paper context

Traditional healers are at increased risk of HIV acquisition through occupational exposure during patient treatment. We will test two implementation strategies (one healer led, one health care provider led) designed to increase uptake and correct use of PPE to reduce blood exposure. If traditional healers prove to be effective educators, we hope to scale up our intervention across South Africa to reach the more than 200,000 practicing healers.

## Appendix

**PPE Training Content Fidelity Evaluation**.

Directions: Score each measure with a value between 0–10 (0 is assigned if the topic is not discussed, 10 for discussed fully); total score is out of 100

1. Delivered information about the definition of PPE.

Score ________

2. Provided information about when to use PPE.

Score ________

3. Provided information about what types of PPE to use in different circumstances.

Score ________

4. Provide information about the appropriate fit of PPE.

Score ________

5. Provide do’s and don’ts of glove use.

Score ________

6. Provided information about don PPE.

Score ________

7. Provided information about how and where to remove PPE.

Score ________

8. Provided information about how to safely store used PPE.

Score ________

9. Provided information about hand hygiene.

Score ________

10. Provided instruction and feedback during PPE practice session.

Score ________

TOTAL SCORE FOR TRAINING SESSION _______/100

**PPE scale measures among traditional healers**

A. Knowledge (True/False/Do Not Know)

1. A person can get HIV by getting an injection with a needle that was already used on someone else.
2. A person can get HIV by sharing blades.
3. A person can get HIV from mosquito bites.
4. A person can get HIV by sharing forks, spoons or cups with a person who has HIV.
5. A person can get HIV from a curse
6. Using gloves during injections will prevent HIV transmission.

B.PPE Motivation (5-point Likert scale, strongly disagree to strong agree)

1. I believe I am at high risk of HIV infection.
2. I believe PPE will prevent me from contracting HIV from my patients during treatments.

C.PPE Self-efficacy (5-point Likert scale, strongly disagree to strong agree)

1. I believe I can use PPE every time I conduct an injection.
  **Table:**
  D. Assessment of PPE Skills at the completion of the training sessions			
  **Hand Hygiene Checklist**			
  Score each method of hand hygiene based on the tasks indicated: done – 1; not done or done incorrectly – 0; not applicable or not assessed – N/A.			
  **Method**	**Task**	**Status**	**Notes**
  **Alcohol-Based Hand Rub**	ABHR is appropriate choice of hand hygiene for the situation		
  	Remove inappropriate jewellery		
  	Use enough alcohol rub to thoroughly wet both hands		
  	Spread product over all surfaces of hands (palms, between fingers, fingertips, back of hands and wrists)		
  	Rub hands for at least 15s or until product is dry		
  **Alcohol-Based Hand Rub Subtotal**			
  **Soap and Water**	Remove inappropriate jewellery		
  	Wet hands and apply soap		
  	Lather all surfaces of hands for 15s (palms, between fingers, fingertips, back of hands and wrists)		
  	Rinse all sides of hands under running water		
  	Dry hands with paper towels		
  	Turn off taps with paper towels		
  **Soap and Water Subtotal**			
  **TOTAL SCORE**			
  **Donning Checklist**			
  Indicate the personal protective equipment (PPE) required for the scenario then number them in the table as the user dons each item. Score the use of each item based on the tasks indicated: done – 1; not done or done incorrectly – 0; not applicable or not assessed – N/A.			
  PPE Required:			
  **User’s PPE sequence↓**	**Task**	**Status**	**Notes**
  **Hand Hygiene #____**	Hand Hygiene was performed		
  	The choice of Hand Hygiene was appropriate		
  **Mask #____**	Chosen mask is appropriate face protection for the situation		
  	Place mask over the nose and mouth		
  	Mold metal strip to nose		
  	Secure elastics or ties		
  **Gloves #____**	Remove inappropriate jewellery		
  	Choose correct type of gloves (if available)		
  	Choose well-fitting gloves		
  	Put on gloves (if gown is worn, place gloves over gown cuffs)		
  **Subtotal**			
  **PPE Selection Score** (Add 5 points for each required item that was selected and subtract 5 points for each necessary item that was **not** selected.)			
  **PPE Donning Sequence Score** (No points if any sequence errors. Award 5 points for each required item/step in a perfect sequence – as listed in table.)			
  **TOTAL SCORE**			
  **Doffing Checklist**			
  Score the use of each item based on the tasks indicated: done – 1; not done or done incorrectly – 0; not applicable or not assessed – N/A.			
  Number of ***items*** to be doffed: _________			
  Number of hand hygiene ***steps*** required: ___________			
  **User’s PPE sequence↓**	**Task**	**Status**	**Notes**
  **Gloves #____**	Remove the first glove using glove-to-glove technique		
  	Remove the second glove using skin-to-skin technique, turning gloves inside out		
  	Drop into garbage without touching bin		
  	Discard the gown in appropriate receptacle		
  **Hand Hygiene #____**	Hand Hygiene was performed		
  	The choice of Hand Hygiene was appropriate		
  **Mask #____**	Undo the ties or elastics without touching the front of the mask		
  	Holding the ties or elastics, lift mask away from the face		
  	Discard mask in the garbage		
  **Hand Hygiene #____**	Hand Hygiene was performed		
  	The choice of Hand Hygiene was appropriate		
  **Subtotal**			
  **Doffing Sequence Score** (No points if any sequence errors. Award 5 points for each required item/step in a perfect sequence – as listed in table.)			
  **TOTAL SCORE**			

## References

[1] King R. Collaboration with traditional healers in HIV/AIDS prevention and care in sub-Saharan Africa: a literature review. UNAIDS Best Practices Collection; 2000. Available from: http://data.unaids.org/Publications/IRC-pub01/jc299-tradheal_en.pdf

[2] Audet CM, Ngobeni S, Graves E, et al. Mixed methods inquiry into traditional healers’ treatment of mental, neurological and substance abuse disorders in rural South Africa. PLoS One. 2017;12:e0188433. PubMed PMID: 29261705; PubMed Central PMCID: PMCPMC5736181. 29261705 10.1371/journal.pone.0188433 PMC5736181

[3] Audet CM, Ngobeni S, Wagner RG. Traditional healer treatment of HIV persists in the era of ART: a mixed methods study from rural South Africa. BMC Complement Altern Med. 2017 8 30;17:434. PubMed PMID: 28854905; PubMed Central PMCID: PMCPMC5577748. 28854905 10.1186/s12906-017-1934-6 PMC5577748

[4] Moshabela M, Bukenya D, Darong G, et al. Traditional healers, faith healers and medical practitioners: the contribution of medical pluralism to bottlenecks along the cascade of care for HIV/AIDS in Eastern and Southern Africa. Sex Transm Infect. 2017 7;93. PubMed PMID: 28736393; PubMed Central PMCID: PMCPMC5739844:e052974. 28736393 10.1136/sextrans-2016-052974 PMC5739844

[5] Moshabela M, Pronyk P, Williams N, et al. Patterns and implications of medical pluralism among HIV/AIDS patients in rural South Africa. AIDS Behav. 2011 5;15:842–11. PubMed PMID: 20628898. 20628898 10.1007/s10461-010-9747-3 PMC4790116

[6] Republic of South Africa. Traditional HealthPractitioners Act, 2007. Vol. 511. Cape Town. 10 January 2008.https://www.gov.za/sites/default/files/gcis_document/201409/a22-07.pdf.

[7] Audet CM, Ngobeni S, Mkansi M, et al. Occupational hazards of traditional healers in rural South Africa: bloodborne pathogen exposures and risk of HIV transmission. Late Breaker Poster Presentation presented at: 22nd International AIDS Conference; 2018 7 23–27; Amsterdam, Netherlands.

[8] Oswald IH. Are traditional healers the solution to the failures of primary health care in rural Nepal? Soc Sci Med (1982). 1983;17:255–257. PubMed PMID: 6857288. 10.1016/0277-9536(83)90327-1 6857288

[9] Green EC. Engaging Indigenous African healers in the prevention of AIDS and STDs. In: Hahn R, editor. Anthropology in public health. Oxford: Oxford University press; 1999. p. 63–83.

[10] Lin J, Puckree T, Mvelase TP. Anti-diarrhoeal evaluation of some medicinal plants used by Zulu traditional healers. J Ethnopharmacol. 2002 1;79:53–56. PubMed PMID: 11744295. 11744295 10.1016/s0378-8741(01)00353-1

[11] Louw CA, Regnier TJ, Korsten L. Medicinal bulbous plants of South Africa and their traditional relevance in the control of infectious diseases. J Ethnopharmacol. 2002 10;82:147–154. PubMed PMID: 12241989. 12241989 10.1016/s0378-8741(02)00184-8

[12] Ssali A, Butler LM, Kabatesi D, et al. Traditional healers for HIV/AIDS prevention and family planning, Kiboga District, Uganda: evaluation of a program to improve practices. AIDS Behav. 2005 12 9;485–493. PubMed PMID: 16249945. DOI:10.1007/s10461-005-9019-9 16249945

[13] Mills E, Cooper C, Seely D, et al. African herbal medicines in the treatment of HIV: Hypoxis and Sutherlandia. An overview of evidence and pharmacology. Nutr J. 2005;4:19. PubMed PMID: 15927053; PubMed Central PMCID: PMC1156943. 15927053 10.1186/1475-2891-4-19 PMC1156943

[14] Madamombe I. Traditional healers boost primary health care: reaching patients missed by modern medicine. Afr Renewal. 2006;19:10.

[15] Rudolph MJ, Ogunbodede EO, Mistry M. Management of the oral manifestations of HIV/AIDS by traditional healers and care givers [Clinical Trial]. Curationis. 2007 3;30:56–61. PubMed PMID: 17515317. 17515317 10.4102/curationis.v30i1.1051

[16] Furin J. The role of traditional healers in community-based HIV care in rural Lesotho [Research Support, Non-U.S. Gov’t]. J Community Health. 2011 10;36:849–856. PubMed PMID: 21374087. 21374087 10.1007/s10900-011-9385-3

[17] Zuma T, Wight D, Rochat T, et al. The role of traditional health practitioners in Rural KwaZulu-Natal, South Africa: generic or mode specific? BMC Complement Altern Med. 2016 8 22;16:304. PubMed PMID: 27549895; PubMed Central PMCID: PMCPMC4994274. 27549895 10.1186/s12906-016-1293-8 PMC4994274

[18] Smyth A, Martin M, Cairns J. South Africa’s health. Traditional healers may cause dangerous delays. BMJ (Clinical Research Ed). 1995 10 7;311:948. PubMed PMID: 7580571; PubMed Central PMCID: PMC2550941. 10.1136/bmj.311.7010.948 PMC2550941 7580571

[19] Audet CM, Blevins M, Rosenberg C, et al. Symptomatic HIV-positive persons in rural Mozambique who first consult a traditional healer have delays in HIV testing: a cross-sectional study. J Acquired Immune Deficiency Syndromes (1999). 2014;66:e80–86. PubMed PMID: 24815853; PubMed Central PMCID: PMC4077983. 10.1097/QAI.0000000000000194 PMC4077983 24815853

[20] Kumakech E, Achora S, Berggren V, et al. Occupational exposure to HIV: a conflict situation for health workers [Research Support, Non-U.S. Gov’t]. Int Nurs Rev. 2011 12;58:454–462. PubMed PMID: 22092324. 22092324 10.1111/j.1466-7657.2011.00887.x

[21] Joyce M, Kuhar D, Brooks J. Notes from the field: occupationally acquired HIV infection among health care workers—United States, 1985–2013. Morbidity and Mortality Weekly Report. Atlanta, GA: Centers for Disease Control and Prevention; 2015. p. 1245–1246. PMC4646046 25577991

[22] Mbaisi EM, Ng’ang’a Z, Wanzala P, et al. Prevalence and factors associated with percutaneous injuries and splash exposures among health-care workers in a provincial hospital, Kenya, 2010. Pan Afr Med J. 2013;14:10. PubMed PMID: 23504245; PubMed Central PMCID: PMCPMC3597860. 23504245 10.11604/pamj.2013.14.10.1373 PMC3597860

[23] Anagaw B, Shiferaw Y, Anagaw B, et al. Seroprevalence of hepatitis B and C viruses among medical waste handlers at Gondar town Health institutions, Northwest Ethiopia. BMC Res Notes. 2012;5:55. PubMed PMID: 22264306; PubMed Central PMCID: PMCPMC3274440. 22264306 10.1186/1756-0500-5-55 PMC3274440

[24] Rossouw TM, Van Rooyen M, Louw JM, et al. Blood-borne infections in healthcare workers in South Africa. S Afr Med J. 2014 11;104:732–735. PubMed PMID: 25909108. 25909108 10.7196/samj.8518

[25] Audet CM, Blevins M, Moon TD, et al. HIV/AIDS-related attitudes and practices among traditional healers in Zambezia Province, Mozambique. J Altern Complement Med. 2012;18:1–9. 23171035 10.1089/acm.2011.0682 PMC3513988

[26] Peters EJ, Immananagha KK, Essien OE, et al. Traditional healers’ practices and the spread of HIV/AIDS in south eastern Nigeria. Trop Doct. 2004 4;34:79–82. PubMed PMID: 15117130. 15117130 10.1177/004947550403400206

[27] Wojcicki JM, Kankasa C, Mitchell C, et al. Traditional practices and exposure to bodily fluids in Lusaka, Zambia. Trop Med Int Health. 2007 1;12:150–155. PubMed PMID: 17207159. 17207159 10.1111/j.1365-3156.2006.01760.x

[28] Scorgie F, Beksinska M, Chersich M, et al. “Cutting for love”: genital incisions to enhance sexual desirability and commitment in KwaZulu-Natal, South Africa. Reprod Health Matters. 2010 1 01;18:64–73. 20541085 10.1016/S0968-8080(10)35500-5

[29] Jolles F, Jolles S. Zulu ritual immunisation in perspective. Africa. 2011;70:229–248. 18360967

[30] Díaz-Guio DA, Ricardo-Zapata A, Ospina-Velez J, et al. Cognitive load and performance of health care professionals in donning and doffing PPE before and after a simulation-based educational intervention and its implications during the COVID-19 pandemic for biosafety. Le Infezioni Med. 2020 6 1;28:111–117. PubMed PMID: 32532947. 32532947

[31] Castro-Sánchez E, Cm ACADP, Me WL, et al. Evaluation of a Personal Protective Equipment (PPE) Support Programme (‘PPE Helpers’) for staff during the Covid-19 pandemic in London. J Hosp Infect. 2020;109:68–77. PubMed PMID: 33307145; PubMed Central PMCID: PMCPMC7722521. 33307145 10.1016/j.jhin.2020.12.004 PMC7722521

[32] Christensen L, Rasmussen CS, Benfield T, et al. Trial of instructor-led training versus video lesson in training health care providers in proper donning and doffing of personal protective equipment. Disaster Med Public Health Prep. 2020 8;14:514–520. PubMed PMID: 32223776; PubMed Central PMCID: PMCPMC7156576. 32223776 10.1017/dmp.2020.56 PMC7156576

[33] Barratt R, Wyer M, Hor SY, et al. Medical interns’ reflections on their training in use of personal protective equipment. BMC Med Educ. 2020 9 23;20:328. PubMed PMID: 32967669; PubMed Central PMCID: PMCPMC7509499. 32967669 10.1186/s12909-020-02238-7 PMC7509499

[34] Kwon JH, Burnham CD, Reske KA, et al. Assessment of healthcare worker protocol deviations and self-contamination during personal protective equipment donning and doffing. Infect Control Hosp Epidemiol. 2017 9;38:1077–1083. PubMed PMID: 28606192; PubMed Central PMCID: PMCPMC6263164. 28606192 10.1017/ice.2017.121 PMC6263164

[35] Luong Thanh BY, Laopaiboon M, Koh D, et al. Behavioural interventions to promote workers’ use of respiratory protective equipment. Cochrane Database Syst Rev. 2016 12 7;12:Cd010157. PubMed PMID: 27925149; PubMed Central PMCID: PMCPMC6464013 non‐profit organization, to conduct this Cochrane review. Bao Yen Luong Thanh: None known. Pornpun Sakunkoo: None known. David Koh: None known. Hla Moe: None known. 27925149 10.1002/14651858.CD010157.pub2 PMC6464013

[36] Salway RJ, Williams T, Londono C, et al. Comparing training techniques in personal protective equipment use. Prehosp Disaster Med. 2020 8;35:364–371. PubMed PMID: 32390583. 32390583 10.1017/S1049023X20000564

[37] Ben-Sasson A, Lior Y, Krispel J, et al. Peer-teaching cardiac ultrasound among medical students: a real option. PLoS One. 2019;14:e0212794. 30917143 10.1371/journal.pone.0212794 PMC6436682

[38] Tejos R, Crovari F, Achurra P, et al. Video-based guided simulation without peer or expert feedback is not enough: a randomized controlled trial of simulation-based training for medical students. World J Surg. 2021 1;45:57–65. PubMed PMID: 32892271. 32892271 10.1007/s00268-020-05766-x

[39] Ten Cate O, Durning S. Peer teaching in medical education: twelve reasons to move from theory to practice. Med Teach. 2007 1 01;29:591–599. 17922354 10.1080/01421590701606799

[40] Centers for Disease Control and Prevention. Exposure to blood: what healthcare personnel need to know. In: National Center for Infectious Diseases Dohqpadovh, editor. Atlanta, USA: CDC; 2003. https://www.cdc.gov/hai/pdfs/bbp/exp_to_blood.pdf.

[41] Kwok YL, Gralton J, McLaws ML. Face touching: a frequent habit that has implications for hand hygiene. Am J Infect Control. 2015 2;43:112–114. PubMed PMID: 25637115. 25637115 10.1016/j.ajic.2014.10.015 PMC7115329

[42] Gomez-Olive FX, Angotti N, Houle B, et al. Prevalence of HIV among those 15 and older in rural South Africa. AIDS Care. 2013;25:1122–1128. PubMed PMID: 23311396; PubMed Central PMCID: PMCPMC3778517. 23311396 10.1080/09540121.2012.750710 PMC3778517

[43] Occupational Safety and Health Administration. Personal protective equipment New York. New York; 2004 [cited 2018 625]. Available from: https://www.osha.gov/Publications/osha3151.pdf

[44] Leow JJ, Groen RS, Bae JY, et al. Scarcity of healthcare worker protection in eight low- and middle-income countries: surgery and the risk of HIV and other bloodborne pathogens. Trop Med Int Health. 2012 3;17:397–401. PubMed PMID: 22035344. 22035344 10.1111/j.1365-3156.2011.02909.x

[45] Federal Drug Administration. Personal protective equipment for infection control, 2018. Silver Spring, MD: U.S. Food and Drug Administration; 2018 [cited 2018 629]. Available from: https://www.fda.gov/medicaldevices/productsandmedicalprocedures/generalhospitaldevicesandsupplies/personalprotectiveequipment/default.htm

[46] Audet CM, Gobbo E, Sack DE, et al. Traditional healers use of personal protective equipment: a qualitative study in rural South Africa. BMC Health Serv Res. 2020;20:655. PubMed PMID: 32669101; PubMed Central PMCID: PMCPMC7362457. 32669101 10.1186/s12913-020-05515-9 PMC7362457

[47] Curran GM, Bauer M, Mittman B, et al. Effectiveness-implementation hybrid designs: combining elements of clinical effectiveness and implementation research to enhance public health impact. Med Care. 2012;50:217–226. PubMed PMID: 22310560. 22310560 10.1097/MLR.0b013e3182408812 PMC3731143

[48] Gertler M, Loik S, Kleine C, et al. [West Africa Ebola outbreak - immediate and hands-on formation: the pre-deployment training program for frontline aid workers of the German Red Cross, other aid organizations, and the German Armed Forces, Wuerzburg, Germany 2014/15]. Bundesgesundheitsblatt Gesundheitsforschung Gesundheitsschutz. 2018 4;61:394–403. PubMed PMID: 29480365. 29480365 10.1007/s00103-018-2710-6

[49] CDC. Personal Protective Equipment (PPE) in Healthcare settings Atlanta, GA2010. [cited 2019 327]. Available from: https://www.cdc.gov/hai/prevent/ppe_train.html

[50] Staff Training: How to Meet OSHARequirements. J Oncol Pract. 2005 Nov;1(4):175-176. doi:10.1200/JOP.2005.1.4.175. PMID: 29442589; PMCID: PMC2794563 PMC2794563 29442589

[51] Phin NF, Rylands AJ, Allan J, et al. Personal protective equipment in an influenza pandemic: a UK simulation exercise. J Hosp Infect. 2009 1;71:15–21. PubMed PMID: 19013670. 19013670 10.1016/j.jhin.2008.09.005

[52] Carroll C, Patterson M, Wood S, et al. A conceptual framework for implementation fidelity. Implement Sci. 2007 11 30;2:40. PubMed PMID: 18053122; PubMed Central PMCID: PMCPMC2213686. 18053122 10.1186/1748-5908-2-40 PMC2213686

[53] Von Thiele Schwarz U, Hasson H, Lindfors P. Applying a fidelity framework to understand adaptations in an occupational health intervention. Work (Reading, Mass). 2015 6 5;51:195–203. PubMed PMID: 24594534. 10.3233/WOR-141840 24594534

[54] Amico KR. A situated-Information Motivation Behavioral Skills Model of Care Initiation and Maintenance (sIMB-CIM): an IMB model based approach to understanding and intervening in engagement in care for chronic medical conditions. J Health Psychol. 2011 10;16:1071–1081. PubMed PMID: 21459919. 21459919 10.1177/1359105311398727

[55] Williams CK, Carnahan H. Development and validation of tools for assessing use of personal protective equipment in health care. Am J Infect Control. 2013;41:28–32. 22704736 10.1016/j.ajic.2012.01.027

[56] Haghighat R. The development of the Brief Social Desirability Scale (BSDS). Eur J Psychol. 2007 11 29;3. DOI:10.5964/ejop.v3i4.417

[57] Sterne JA, White IR, Carlin JB, et al. Multiple imputation for missing data in epidemiological and clinical research: potential and pitfalls. BMJ (Clinical Research Ed). 2009;338:b2393. PubMed PMID: 19564179; PubMed Central PMCID: PMCPMC2714692. 10.1136/bmj.b2393 PMC2714692 19564179

[58] VanderWeele TJ. Mediation analysis: a practitioner’s guide. Annu Rev Public Health. 2016;37:17–32. PubMed PMID: 26653405. 26653405 10.1146/annurev-publhealth-032315-021402

